# Exposure in Horses to Human Tick-Borne Relapsing Fever Agent *Borrelia persica*, Israel, 2025

**DOI:** 10.3201/eid3205.251283

**Published:** 2026-05

**Authors:** Dor Shwartz, Lior Haras, Yaarit Nachum-Biala, Sharon Tirosh-Levy, Amir Steinman, Gad Baneth

**Affiliations:** Hebrew University of Jerusalem, Koret School of Veterinary Medicine, Rehovot, Israel

**Keywords:** *Borrelia persica*, bacteria, bacterial infection, horses, tick-borne relapsing fever, vector-borne infections, zoonoses, Israel

## Abstract

Human tick-borne relapsing fever caused by *Borrelia persica* is common in western Asia. A survey of 301 horses in Israel revealed 9.96% seropositivity toward *B. persica* antigens; 1 horse (0.33%) was also PCR positive for *B. persica* DNA. Phylogenetic analysis supported a transmission cycle involving ticks, humans, and horses.

Tick-borne relapsing fever (TBRF) is a human and veterinary illness caused by spirochetes of the genus *Borrelia* (*1*). *Borrelia persica*, transmitted by the argasid tick *Ornithodoros tholozani*, is the primary cause of TBRF in Israel and affects persons in other parts of the eastern Mediterranean basin and Asia (*2*–*4*). Relapsing fever in humans is characterized by episodes of recurrent fever, lethargy, and headache; an up to 10% mortality rate in untreated patients has been documented (*5*). TBRF caused by *B. persica* in dogs and cats is associated with fever, lethargy, anorexia, anemia, and thrombocytopenia and can be fatal (*6*). Several species of wild animals are known potential reservoirs for *B. persica* spirochetes, including the red fox (*Vulpes vulpes*), golden jackal (*Canis aureus*), European badger (*Meles meles*), and some rodent species (*7*–*9*). In contrast to *Borrelia* spp. that cause Lyme disease in horses, for which high seroprevalence has been reported in several countries (*10*), equine TBRF is poorly documented. One case of equine abortion has been reported in a mare from California infected with either *B*. *parkeri* or *B*. *turicatae*, which could not be distinguished (*10*,*11*). We tested samples from horses from throughout Israel for *B. persica* seroreactivity.

## The Study

We collected samples from 301 clinically unremarkable horses from 27 stables throughout Israel, representing the geographic distribution of the equine population, and from 12 horses living in a nonendemic area for TBRF in southern Israel, where *O. tholozani* ticks have not been reported (*7*), which served as negative controls. Of the 301 horses, 1 (0.33%) was positive for *B. persica* DNA by using real-time PCR amplification, confirmed by sequencing of the *flaB* and *glpQ* genes, as described previously (*9*). Thirty (9.96%) horses were seroreactive against *B. persica* antigen by ELISA testing. 

The ELISA assay was developed and performed as previously described for dogs and cats (*8*) with adaptation to horses. We extracted crude antigen from 1 L of *B. persica* culture containing 10^7^ spirochetes/mL and lysed by sonication. We coated each well with 0.7 µg of antigen. We performed blocking by using 5% skimmed milk powder dissolved in phosphate-buffered saline (PBS) (Merck KGaA, https://www.emdgroup.com) and incubated the plates overnight at 4°C. We diluted the tested serum 1:500 in PBS with 0.1% Tween 20 (PBS-T) and 2% fetal bovine serum. We incubated serum bound antibodies for 1 hour at 37°C with horseradish peroxide-conjugated goat anti-horse IgG (ABCAM, https://www.abcam.com) diluted 1:50,000 in PBS-T and 2% fetal bovine serum. We read each plate when the absorbance (λ = 405 nm) of the positive reference serum reached an optical density (OD) value of 1.1. We determined a cutoff value of 0.35 OD by adding 3 SEs to the mean absorbance of serum from the negative control group. Serum from the horse found positive for *B. persica* by real-time PCR and sequencing was used as a positive control.

**Figure 1:**
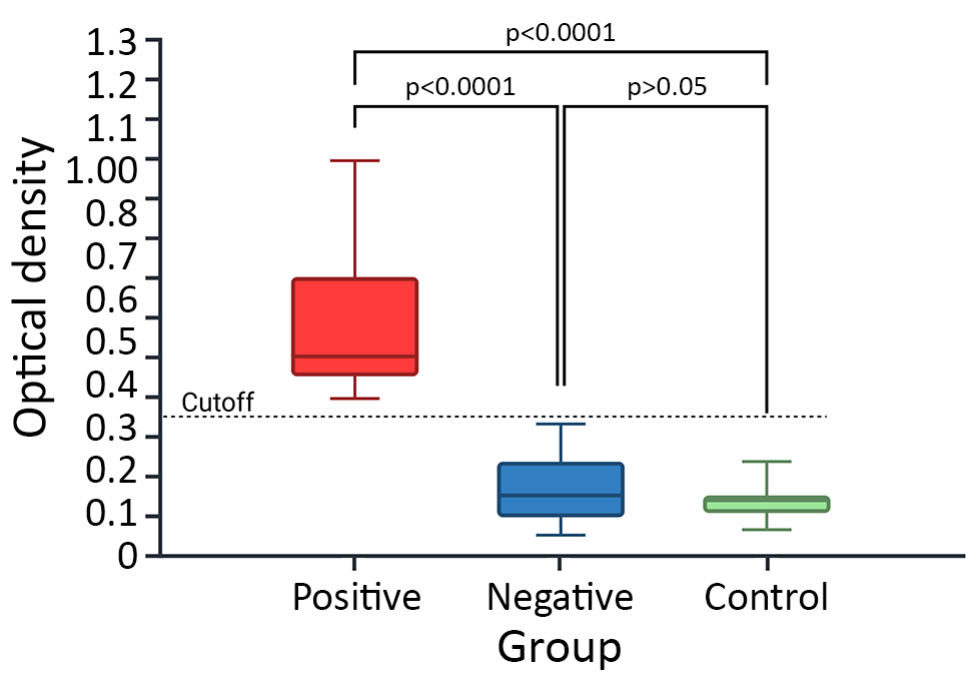
Comparative analysis of serologic responses of horses exposed to human tick-borne relapsing fever agent *Borrelia persica*, Israel, 2025. Box-and-whisker plot demonstrates optical density measurements from seropositive (blue), seronegative (red), and negative control (green) horses. The dashed line represents the established cutoff value (optical density = 0.35). Statistical significance was determined using a Kruskal–Wallis test followed by Dunn multiple comparison test. Results significantly distinguish between the seropositive horses compared with the seronegative and control groups, whereas the seronegative group did not significantly differ from the negative control. Horizontal lines within boxes represent median values; box tops and bottoms indicate the upper and lower quartiles; error bars represent ranges. p values are indicated.

The median serologic OD was 0.472 for the seropositive, 0.145 for the seronegative, and 0.138 for the negative control horses (Figure 1). We found significant differences between the 3 groups (Kruskal-Wallis H = 64.72; p<0.0001). Posthoc Dunn tests demonstrated that seropositive horses had a higher OD than both seronegative (adjusted p<0.0001; mean rank difference = 156.98) and negative control (adjusted p<0.0001; mean rank difference = 182.00) groups. We did not observe a significant difference between seronegative and negative control groups (adjusted p = 1.000; mean rank difference = 25.02) (Figure 1). Seropositive horses were significantly older than seronegative ones (mean +SD 13.8 +6.3 years for seropositive vs. 10.8 +5.4 years for seronegative; Mann-Whitney U = 2,633, p = 0.009). Furthermore, we found age was a significant independent predictor of TBRF seropositivity with an odds ratio (OR) of 1.09 per year (95% CI 1.03–1.17; p = 0.007). Seropositivity did not significantly differ according to sex (χ^2^ = 2.32; p = 0.313) or housing type (χ^2^ = 2.01; p = 0.367).

**Figure 2:**
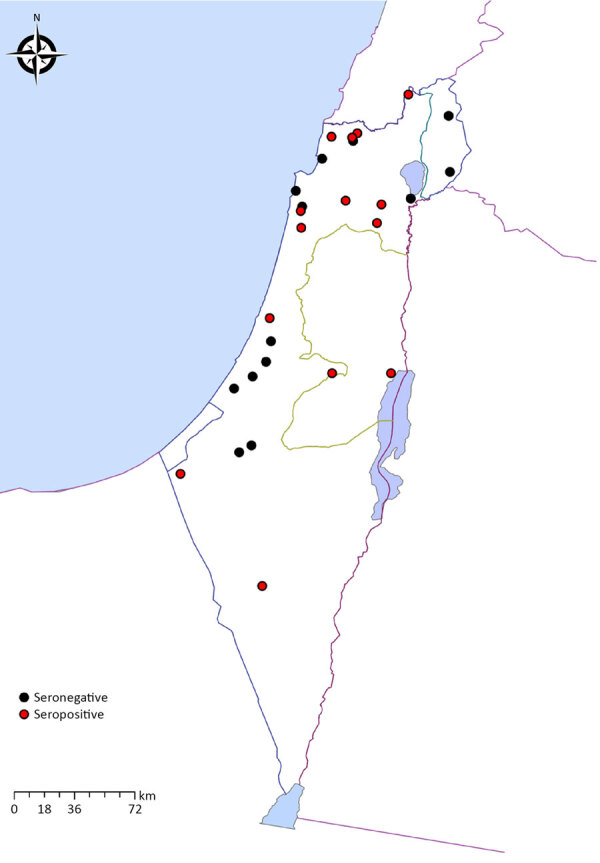
Geographic distribution of sampled horses in study of horses exposed to human tick-borne relapsing fever agent *Borrelia persica* in Israel that indicates the seroprevalence. Red circles represent the locations of seropositive horses; black circles denote seronegative horse locations.

The PCR-positive horse was also found to be seropositive (OD 1.04). It was a 10-year-old gelding from a stall in the city of Ramat Gan, Israel. Sequencing analysis of a 272-bp segment of the *flaB* and a 224-bp segment of the *glpQ* gene fragments from the positive horse revealed 100% identity and coverage to a *B. persica* amplified from a human (GenBank accession no. DQ679907.1 [*flaB*]) and from an *O*. *tholozani* tick (GenBank accession no. HM161658.1 [*glpQ*]). Seropositive horses resided in 15 different locations (Figure 2). All positive horses were adults with a median age of 13 years (range 3–38 years); there were 18 geldings and 12 mares.

**Figure 3:**
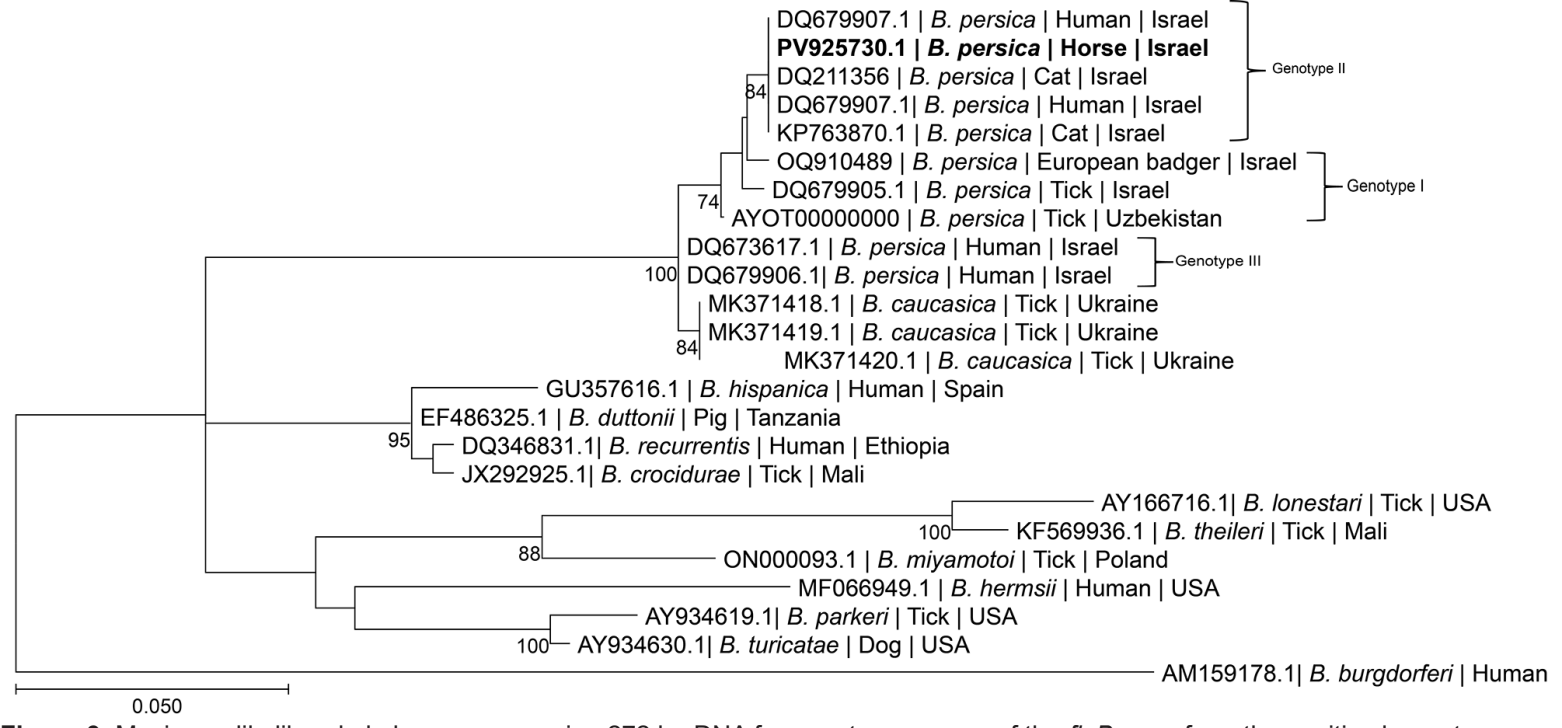
Maximum-likelihood phylogram comparing 272 bp DNA fragment sequences of the *flaB* gene from the positive horse to sequences from other *Borrelia persica* and other *Borrelia* spp. GenBank accession numbers in a study of *B. persica* exposed horses in Israel. Bold indicates new sequence derived from this study. *B. persica* genotypes are noted at right. GenBank accession number, species of infected host, and country of origin is provided for each sequence. The Tamura 3-parameter model was used in the construction of this phylogram with bootstrap performed on 1,000 replicates; values >70% are indicated. Scale bar represents the number of nucleotide substitutions per site; branch numbers indicate bootstrap support values, expressed as percentages.

**Figure 4:**
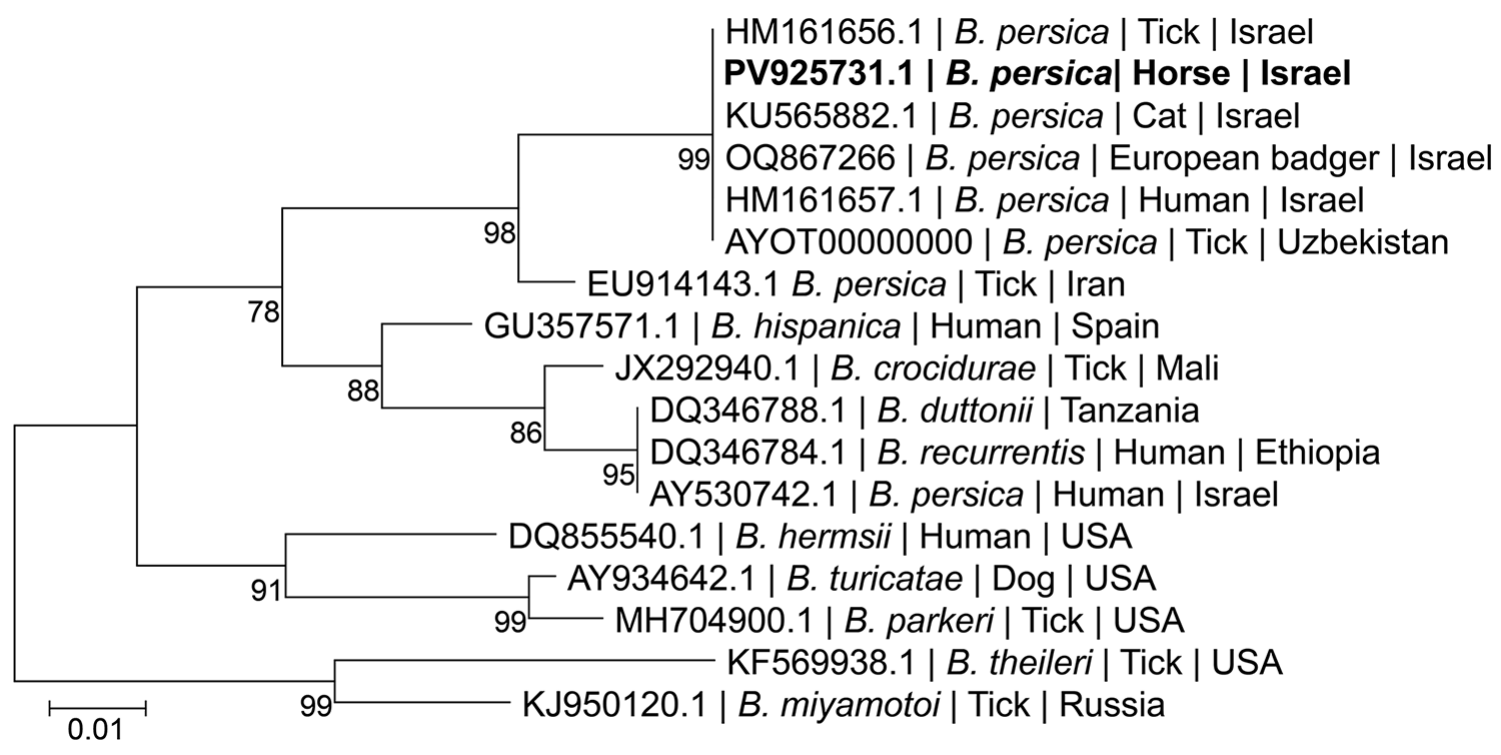
Maximum-likelihood phylogram comparing 224 bp DNA fragment sequences of the *glpQ* gene from the positive horse sequences from other *B. persica* and other *Borrelia* spp. GenBank accessions. Bold indicates new sequence derived from this study . GenBank accession number, species of infected host, and country of origin is provided for each sequence. The Tamura 3-parameter model was used in the construction of this phylogram with bootstrap performed on 1,000 replicates; values >70% are indicated. Scale bar represents the number of nucleotide substitutions per site; branch numbers indicate bootstrap support values, expressed as percentages.

Phylogenetic analysis on the basis of a 272-bp segment of the *flaB* gene sequence (Figure 3) revealed that the *B. persica* sequence from the positive horse clustered together with a *B. persica* genotype II sequence previously amplified from a human in Israel (GenBank DQ679907.1) (*12*). All *B. persica* sequences clustered separately from other Old World relapsing fever *Borrelia* spp. that are transmitted by ixodid ticks, including *B. lonestari* and *B. theileri*, which clustered together, but separately from *B. miyamotoi*, which clustered with *B. hermsii*. Phylogenetic analysis on the basis of a 224-bp segment of the *glpQ* gene (Figure 4) also revealed that the sequence from the *B. persica*–positive horse clustered with other *B. persica* sequences from a human, a cat, a European badger, and an *O. tholozani* tick and with the corresponding sequence of the only published whole-genome reference of *B. persica* from an *Ornithodoros papillipes* tick in Uzbekistan (GenBank accession no. AYOT00000000).

## Conclusions

This study describes a survey of TBRF *Borrelia* spp. in horses. Furthermore, it introduces a new ELISA optimized for horse serum to detect antibodies against *B. persica*, enabling seroepidemiologic investigations. Our findings indicate an unexpectedly high seroprevalence (9.96%) among clinically healthy horses, suggesting high exposure to *B. persica* and to infected *O. tholozani* ticks. *B. persica* DNA was detectable in 1 of the seropositive horses, which indicates that horses are not only exposed to this spirochete but also might develop a circulating infection. The horses included in the study were all apparently healthy, but, as is the case for humans, dogs, and cats, illness might develop after infection. Furthermore, horses might serve as potential reservoir hosts for TBRF, which could then be transmitted to humans and other animals.

Israel and the surrounding region are not endemic for Lyme borreliosis, and no autochthonous infections have been reported in humans or animals in Israel to our knowledge. Therefore, serologic cross-reactivity with a Lyme borreliosis agent is unlikely in the studied horses. Nevertheless, the potential occurrence of other relapsing fever species besides *B. persica* in Israel is possible (*13*). The *glpQ* phylogram displays variation in 2 *B. persica* sequences (GenBank accession nos. EU914143.1 [tick] and AY530742.1 [human]) that were amplified in Israel. Those sequences share <95% identity with other *B. persica glpQ* sequences deposited into GenBank and could possibly belong to different species.

Increasing age was associated with seropositivity, which aligns with prolonged or cumulative exposure to tick vectors during the horse lifespan. Neither horse sex nor housing conditions was associated with seropositivity, indicating exposure regardless of management practices.

The phylogenetic analyses we report support a potential shared transmission cycle of *B. persica* involving horses, humans, and *O. tholozani* ticks, and suggesting a potential zoonotic risk associated with infected equine populations. Horses might serve as reservoirs and sentinels for human infection with *B. persica*, and One Health approach strategies should be implemented to monitor and manage risks posed by this pathogen.
